# O-22 - SUSPECTED HCID ASSESSMENT, TESTING AND ISOLATION: EXPLORING A CONUNDRUM

**DOI:** 10.1093/pubmed/fdag046.019

**Published:** 2026-07-09

**Authors:** Dr Christopher Redman, Mary Stewart, Dr Oliver Koch, Professor Catherine Moore, Dr Kim Marsh, Dr Kate Templeton, Professor Rory Gunson, Mark Traherne, Sophie French, Dr Iain Kennedy, Carrie-Anne Gibb, Dr Kate Smith

**Affiliations:** Zoonoses, Emerging Infections and Borders team, Public Health Scotland; Public Health Department, NHS Fife; Regional Infectious Disease Unit, NHS Lothian; Public Health Microbiology Division, Public Health Scotland; Viral Pathogens, Infectious Respiratory Disease Team, Public Health Scotland; Scottish National VHF Test Service, NHS Lothian; West of Scotland Specialist Virology Centre, NHS Greater Glasgow and Clyde; Scottish Ambulance Service, NHS Scotland; Antimicrobial Resistance and Healthcare Associated Infections, NHS National Services Scotland; Public Health Protection Unit, NHS Greater Glasgow and Clyde; Zoonoses, Emerging Infections and Borders team, Public Health Scotland; Zoonoses, Emerging Infections and Borders team, Public Health Scotland

## Abstract

**Background:**

In the UK key pathogens are classified as High consequence infectious diseases (HCID) - GOV.UK.). Admission of suspected HCID cases to acute settings can significantly disrupt clinical services, despite many patients not requiring hospital care at the time of assessment. Community-based pathways for HCID assessment and sampling vary in availability across Scotland. Remote and rural geography adds further challenges in securing rapid, reliable test results to enable timely standdown or, rarely, escalation of a confirmed case.

**Objectives:**

To reflect on Scotland’s experience in managing suspected and confirmed HCID cases, consider evidence from other UK nations on the impacts of confirmed HCIDs, and describe strengths and challenges identified by multi-agency partners in assessment, sampling and isolation across acute and community settings.

**Methods:**

Literature review of HCID-related impacts on clinical and public health services in the UK (2010–2026); reflection on HCID pathway activations in Scotland; and engagement with multi-agency partners involved in preparedness and response.

**Results:**

Confirmed HCID cases detected in Scotland include H1N1 (2009), CCHF (2012), Ebola virus disease (2014), SARS-CoV-2 (2020) and mpox clade 2 (2022). HCID pathways are also regularly activated for suspected cases, particularly avian influenza and MERS. A community-based approach—maintaining selected patients at home under a multi-agency risk-assessed plan—may offer a more proportionate, patient-centred model. Risk perception and tolerance vary across partner organisations. Strengthened tools, structures and agreements are needed to support consistent multi-agency decision-making and risk management.

**Conclusions:**

Balancing low-likelihood, high-impact risks remains challenging. Clear, streamlined pathways for sampling—whether in emergency departments, wards or at home—are essential, alongside rapid, reliable diagnostics to enable timely de-escalation or, rarely, escalation. A risk-assessed, multiagency community-supported approach may be appropriate but must also consider the transmission risk to the household and community and the need for safe isolation.

